# The implementation of a quality system in the Dutch GP specialty training: barriers and facilitators; a qualitative study

**DOI:** 10.1186/s12909-017-0947-7

**Published:** 2017-07-21

**Authors:** Nienke Buwalda, Jozé Braspenning, Sanne van Roosmalen, Nynke van Dijk, Mechteld Visser

**Affiliations:** 1 Department of General Practice/Family Medicine, Academic Medical Center-University of Amsterdam, Meibergdreef 9, 1105 AZ Amsterdam, The Netherlands; 2 IQ Scientific Institute for Quality of Healthcare, Radboud University Medical Center, Nijmegen, The Netherlands

## Abstract

**Background:**

Quality assurance programs in medical education are introduced to gain insight into the quality of such programs and to trigger improvements. Although of utmost importance, research on the implementation of such programs is scarce. The Dutch General Practice (GP) specialty training institutes used an implementation strategy to implement a quality system (QS), and we aimed to study the success of this strategy and to learn about additional facilitators and barriers.

**Methods:**

Seventeen structured interviews were conducted with the directors and quality coordinators (QCs) of the eight Dutch GP training institutes. A five-stage process model of implementation was used to structure these interviews and analyze the data. Two researchers analyzed the data with a framework approach.

**Results:**

The strategy supported the institutes in implementing the QS. However, after the introduction of the QS, staff experienced the QS as demanding, although they noticed almost no concrete short-term results. Moreover, they experienced difficulties in integrating the QS into their local situation. Collectively working with the QS and following common deadlines did create a sense of commitment towards each other that appeared to be a true stimulus to the introduction of the QS.

**Conclusions:**

The implementation strategy focused mainly on the introduction of the QS in the GP specialty training, and it was, as such, rather successful. An important barrier concerned the acceptance of the QS and the integration of the QS into local structures, which suggests that there is a need for guidance on the translation of the QS to local contexts. All in all, we recommend more focus on the benefits of a QS.

## Background

Quality assurance and improvement in medical education are of paramount importance, not only for the benefit of medical students and future doctors, but above all for patients [1]. Quality assurance in education involves evaluating and improving activities and processes of both teaching and learning [2, 3], and there are differences in the way organizations manage and assess the quality of their education [4, 5]. To create more uniformity internationally and to stimulate improvement and assure minimum quality standards, the World Federation for Medical Education (WFME) has developed global standards [5, 6]. Organizations worldwide use this framework as a model, for example to establish national and regional accreditation objectives [5]. The framework covers all relevant aspects of basic medical education [7], post-graduate medical education [8] and continuing professional development [9].

Quality standards, such as those of the WFME, can serve as a basis for quality systems (QSs). Organizations use QSs to manage quality in a systematic manner. However, the actual implementation is of crucial importance to the success of a QS: innovation failures are often due to unsuccessful implementation rather than perceived ineffectiveness of the innovation [10]. Although many studies have described the implementation of innovations in health care [11], little is known about the implementation processes of QSs in the field of medical education. Because quality management in medical education is growing, there is a need for understanding the implementation process of such systems to identify the barriers to and enablers of improvement [12–14].

The implementation of a QS starts with the decision to adopt an intervention [15]. Based on theories that consider implementation to be a step-by-step process, Grol and Wensing (2011) proposed a five-stage model to support the design of an implementation strategy [16]. These stages are known as (1) orientation, (2) insight, (3) acceptance, (4) change, and (5) consolidating change. Ideally, the stages proceed as follows: in the first stage, the people involved receive information about the innovation and become aware of, and get interested in, the innovation, in this case: the new QS. The second stage will help them understand the benefits and get prepared, for example by following training to learn more about the innovation. In the third stage, people will develop a positive attitude, get motivated, and will want to get started: they accept the innovation. People move on to the fourth stage when they start to work with the innovation and experience its advantages. In the final stage, they integrate the innovation into their daily work.

**Fig. 1:**
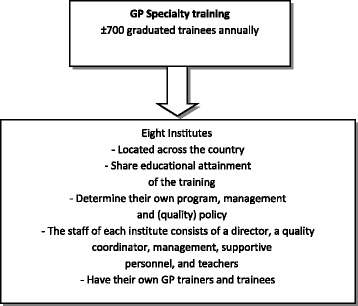
Dutch GP Specialty Training

The Dutch General Practice (GP) specialty training institutes (Fig. 1) developed and implemented a QS, named GEAR, to stimulate quality assurance, quality improvement and collaboration between institutes. Based on the wishes of the institutes, GEAR aimed to combine the virtues of two earlier initiatives in order to provide clear and shared assessment criteria. GEAR would also act as an advisory tool to stimulate the quality of the training [17]. A project team was commissioned by the GP specialty training to develop the new QS. This team consisted of four experts from GP care and quality care. They were advised by a sounding board, made up of a director of an institute, GP trainers, a trainee, an expert in quality management, and six representatives of professional associations. Additionally, the directors of the eight institutes were closely involved in the development of the new system [17].

After listing possible barriers and facilitators, the project team developed an implementation strategy that included four components: (1) the involvement of the directors of training institutes in the development of the new QS (content and process); (2) a web-based, professional, supportive data-entry system including a comprehensive manual; (3) a training program; and (4) a national quality coordinator to support the institutes with improvement activities. Table 1 shows the implementation strategy used for the new QS and its intended effect on the different stages of the model of Grol and Wensing [16]. The aim of this paper is to gain insight into the effect of the strategy and to learn about the additional factors that affect the implementation of a QS in a postgraduate medical specialty training.

**Table 1:** Components of the implementation strategy used for the QS and their intended effects

	1. Involving directors	2. Web-based, professional supportive system	3. Coordinated training program	4. National Quality Coordinator
	Directors decide to develop and use a collective QS, and they are involved in the development of the system	Developers design the system in a systematic and thoughtful manner. There is an informative manual, a web-based system for data management, enthusiastic (organizational) support, and clear national deadlines.	Before launch, there is a presentation including a clear explanation of the purpose of the QS. All quality coordinators (QCs) receive training in how to work with the web-based system. The audit commission receives a 1-day professional training.	A national quality coordinator supports the institutes.
Stage1. Orientation	There is attention for the system before it is put into use. Those involved know the system is coming.		The presentation and training sessions provide the participants with more insight into the relevance of the system.	
Stage 2. Insight		The informative manual will help to prepare the institutes.	QCs are skilled enough.	
Stage 3. Acceptance	Increases the credibility and the commitment to the system.	A professional system will enhance the credibility of the system and create confidence.	Expectations and responsibilities are clear. Involving others in the training sessions leads to more support.	
Stage 4. Change	It is certain that the system will be put into use.	Clear national deadlines ensure that all institutes take the same steps at the same time. In the case of problems, there is support.	QCs are capable of working with the system.	
Stage 5. Consolidating change				The national quality coordinator supports the institutes with implementing improvement plans.

## Methods

### Context

**Fig. 2:**
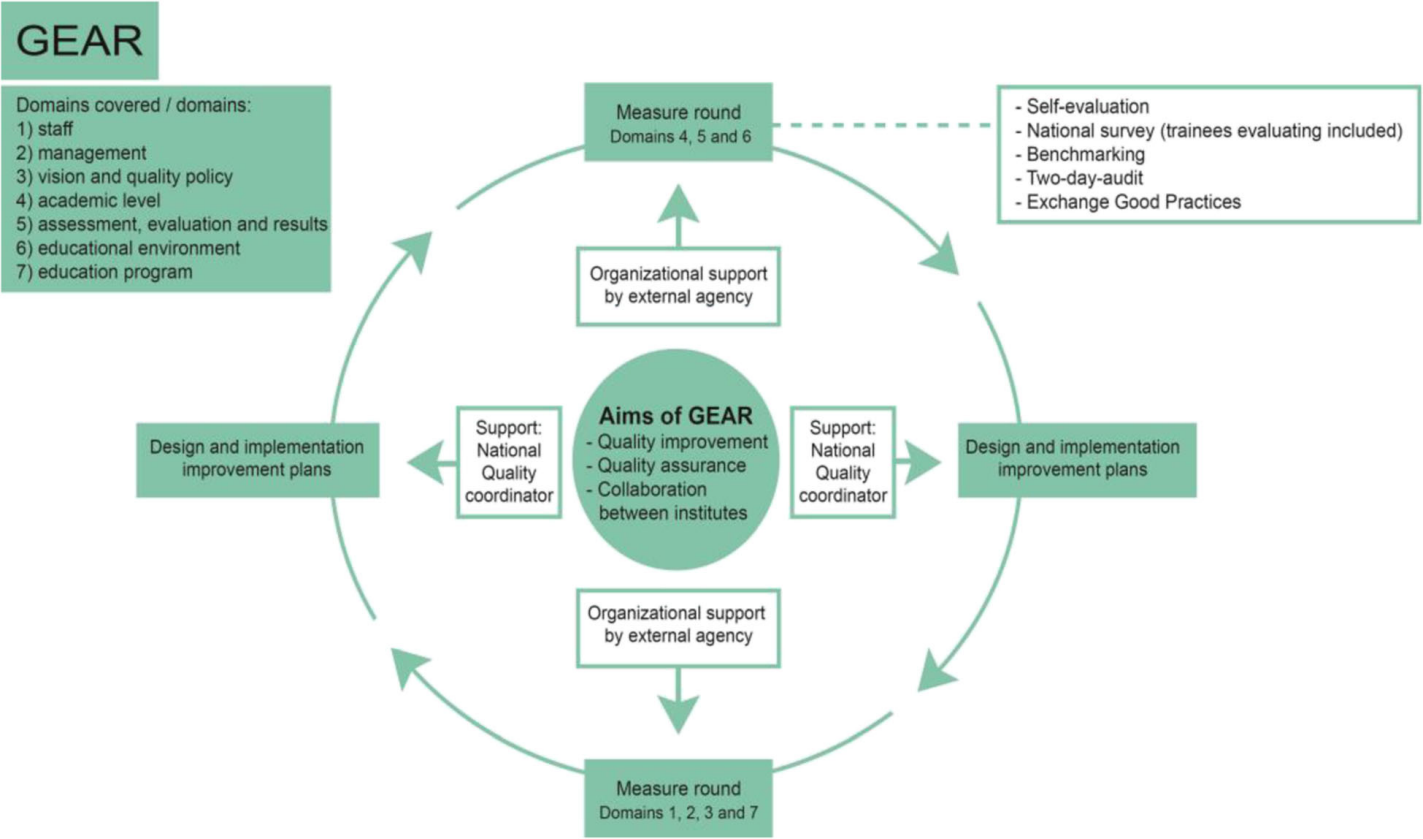
GEAR figure. GEAR assesses the institutes in seven domains. The domains correspond with the WFME standards, but they have been adapted to the GP specialty training. All domains are assessed once every five years. Quantitative and qualitative assessment methods are used. The introduction of the system starts with self-evaluation and involves deadlines to ensure that all institutes take the different steps at the same time. Semi-annual meetings take place to exchange Good Practices. After the measurement round, institutes design and implement improvement plans

The Dutch GP specialty training is a 3-year postgraduate training (Fig. 1). There was a need for one collective and structured system that would serve multiple purposes: quality assurance, quality improvement, and the enhancement of cooperation between the institutes. For this reason, the institutes jointly developed a new QS in 2011. The new QS encompassed self-evaluation, benchmarking among institutes, audits, exchange of good practices, and improvement plans; in addition, a quality coordinator was involved to stimulate the exchange [17] (Fig. 2).

### Participants

To explore the effects of the preparatory strategy, the directors of all GP specialty training institutes were interviewed, as well as one former director (who was involved in the development and the implementation of the system) and eight quality coordinators (QCs) (*n* = 17). The directors were involved in the development of the new QS and tasked with the expansion of the QS at the institutes. The QCs were tasked with coordinating the collection of data at the institutes and were trained to work with the (web-based) QS. Participants were informed that participation was voluntary and that the transcribed interviews would be coded to prevent responses being traceable to individual participants or departments. All participants agreed with this and gave written informed consent.

### Data collection

Semi-structured face-to-face interviews were conducted individually at the interviewee’s training institute from May to August 2013, after the first audit round. One researcher (NB) conducted all the interviews. They were audio-recorded and transcribed verbatim.

### Interview

The interviews were conducted using a semi-structured questionnaire (Table 2). The questionnaire was pilot-tested on two members of one of the GP specialty training institute, which led to a few adjustments. The resulting questionnaire included a topic list that consisted of six categories: (1) general questions, (2) system specific questions, (3) questions about the implementation process, (4) questions about the results, (5) evaluation questions, and (6) questions about the support. These six categories were divided into sub-categories, which included keywords (for example: provision of information, relevance, acceptance, skills) that referred to Grol and Wensing’s five stage model (1) orientation, (2) insight, (3) acceptance, (4) change, and (5) consolidating change) [16]. To get an overall picture, questions about the experiences of staff in the organization were added. In the interview with the QCs, two more questions were added about how they had been informed and instructed.

**Table 2:** Structure of interviews

Category	Sub-category	Summary of the questions per block
1. General	Motivation (vision) Quality policy Quality culture Involvement Expectations Confidence Acceptance	What is the general perception of the need for a quality system, how does it fit into the quality policy and the organization culture, and how do participants view the system?
2. Content	Clearness Relevance Completeness Domains and indicators	How is the system developed? Is it clear, do participants and the staff see its relevance, and is it complete? What is the opinion about the system? Is this shared with the staff?
3. Processes	Provision of information Skills Implementation process (steps) Means Roles in the organization	How is the system introduced at the institute, how does the implementation process proceed, and ho is involved?
4. Products	Costs and benefits	What have been the costs and benefits of the system so far?
5. Process-evaluation	Meaningfulness Incentives and barriers Improvements	What are the experiences of working with the system?
6. Support	Practical Content	How did the participants experience the support while working with the system? Was it enough and what do they need in the future?

### Data analysis

A ‘framework’ approach was used to analyze the data [18]. Upon completion of all interviews, the relevant interview excerpts were identified (NB, SvR) and divided into categories that referred to the theoretical model of implementation [16]. Outcomes were discussed until consensus was reached. Subsequently, the relevant text fragments were electronically coded using MAXqda software to further refine the analysis by grouping similar fragments together and selecting key categories within each stage. The data was also scanned for patterns of similarity or dissimilarity across and within departments and for differences between directors and QCs within the departments.

## Results

We interviewed eight directors (four male and four female), one former director (male) and eight QCs (three male and five female). On average, the interviews lasted 80 min, ranging from 45 to 110 min.

### Five-stage model

This section describes the findings from the interviews grouped per stage of the model of Grol and Wensing [16]. Table 3 gives an overview of the strategies, barriers, and illustrative quotes.

**Table 3:** Strategies, barriers and quotes in each stage

Stage	Strategies	Barriers	Quotes
1. Orientation	Involving directors There was attention for the QS before it was put into use. Those involved in the development (most directors) knew the system was coming. Training program An information meeting beforehand prepared the institutes and helped the participants and staff to gain more insight into the relevance and need of the system.	Seeing no relevance Not ready for the innovation	*“We were well informed” (D1)* *“The outside world must be able to rely on a strong, common system.” (D2)* *“The system could give us more structure to actually work with improvements plans.” (D8)* *“I saw no relevance in using this collective system. I believe all the institutes are able to systematically monitor their quality. Paying attention to quality nationwide is good, but not to assess every institute with the same indicators, because we are all different. Benchmarking with these indicators is not possible.” (QC5)* *“We had little time to set a date for the information meeting. We preferred to start a little bit later with the QS.” (D9)*
2. Insight	Training program Quality coordinators were skilled enough. Professional system Participants thought this system was an improvement compared to the prior systems. The professional appearance with the informative manual helped the institutes to prepare.	Wait-and-see attitude (passive) Not everything is clear Seeing no improvement to the former situation	*“I am curious about what the QS will bring to us. I will wait and see.” (D1)* *“What is the role of the national guidance? I don’t know what their role is.” (QC7)* *“It is such a comprehensive system. For me, it is too much to have an overview.” (D1)* *“I really need our quality coordinator, to be able to work with the system.” (D9)* *“For me, the purpose of the system is collecting data and showing this to the audit commission. I have no more expectations.” (D6)*
3. Acceptance	Involving directors Increased the credibility and the commitment to the system among QCs and staff Professional system Enhanced the credibility of the system and created confidence among participants and staff.	Doubts about / critical towards the system: does the system really assess quality? System does not fit at all institutes It feels imposed	*“The way we can use good practices from other institutes is complicated because every institute works with their own formats and has its own structure. It is not as easy as it looks”. (QC2)* *“The starting point of the system is positive. What can we contribute to others and what can they contribute to us?” (D5)*
4. Change	Involving directors It was certain that the system was put into use. Training program QCs were capable of working with the system. Professional system Clear national deadlines ensured that all institutes made the same steps at the same time. In the case of problems, there was support.	Little space for flexibility Time pressure, tensions because of the deadlines and quantity of tasks Lack of new insights	“*There is no flexibility, so the system is perceived as rigid.”* (D9) “*Finding the right documents and the problem is that sometimes there is no separate document. It is intertwined. To get that out and specify and referencing. That is difficult. So it is quite a job.” (D4)* “*You have to be alert not to overload people. I do have the time for it; it is my job, but most people in the organization don’t.” (QC4)* *“It is a pity that there is little space to argue and to explain why we make conscious choices that differ from [choices underlying] the indicators*.” (QC6)
5. Consolidating change	The institutes received no support from the national QC at the moment of the interviews.	Attention fades	“*It is like a pilot light. When there is attention, it burns again, but after that, it fades away.” (QC5)*

## Orientation

All participants confirmed that they were aware that the QS would be introduced, either because there had been a clear announcement or because they had been involved in the development of the QS, or both. The information meeting in which the system was introduced to the staff was evaluated positively, and some participants said that the meeting had motivated them to use the QS. One QC, however, was not motivated by the information meeting: this QC considered a common QS to be of little use, given the differences between the institutes. The QC would have preferred a separate QS for each individual institute, albeit with national support.

The perceptions concerning the relevance of the QS differed. Some participants mentioned that the QS could be a tool for mutual benefit, that it would enhance uniformity between institutes, and that it would show to the outside world that the GP training institutes were quality-driven. Other participants were more focused on internal aspects. For them, the QS helped to focus on quality in a structured way and served as a mirror to gain insight into processes within their own institute.

Although the institutes prepared for the start of the QS, not all institutes felt ready for it. Some participants mentioned that their institute was not able to invest the time and resources they expected would be necessary. Participants said that both they and their staff members therefore felt resistance to the QS. For some of the institutes, the information meeting proved to be helpful in taking away this resistance: “*One of the developers of the QS came and spoke. She explained and illustrated the system to all the staff, after which the mood became more positive. It was a good decision to introduce the system in this way.” (QC5).*In sum: the strategies proved helpful for some institutes to motivate staff and reduce resistance. During the orientation stage, we also observed two main barriers: (1) not all the participants saw the relevance of the QS, and (2) not all institutes felt ready to implement the new QS.

## Insight

All QCs received professional training, and participants confirmed they gained a better understanding of the QS because of the training. They evaluated the training positively, partly because it offered them an opportunity to meet the QCs of other institutes which created a sense of togetherness. During this training, the QCs were given a comprehensive and informative manual to prepare themselves and others in the institutes, which they said was helpful.

Participants mentioned that the QS was an elegant and well-designed system, that the manual was informative and looked professional, and that the support was provided by a team of professional and experienced advisers. They also indicated that the goal, the meaning and global processes of the QS were fully clarified to them. Still, participants sometimes had to deal with uncertainties (Table 3: “quotes”*)*. This was the case among the directors more often than among the QCs. Additionally, most participants were apprehensive about the system’s benefits for their respective institutes. Some of the participants revealed that they did not explicitly need the QS for receiving insight into strengths and weaknesses of their own institute because, for example, they already had some kind of quality system. Some directors had negative or neutral attitudes towards the importance of the QS. Nevertheless, the new QS was experienced, by and large, as much better than the previous systems.

The training and the professional system (component 2 and 3 of the implementation strategy) helped the participants to work with the QS: they gained a better understanding. However, there were still uncertainties, and the need for a shared QS for their own institute was not always evident. The barriers we observed were: (1) participants had a passive attitude with low expectations, (2) not everything was clear, and (3) some participants did not see the new QS as an improvement to the prior situation.

## Acceptance

Most participants agreed on the goals and assumptions, and on the premise that the GP specialty training institutes, as professional organizations, had to have a collective system. They also accepted the framework of the system, but they did not accept the realization of the system in its entirety. The main criticism on the QS was that it seemed to assess preconditions for quality, instead of quality itself. The participants also doubted the credibility of the benchmarking and the feasibility of exchanging good practices; in addition, they doubted the audit because it was a snapshot of the institute and, therefore not reliable, according to some participants.

Some participants mentioned that the system did not suit their institute: “*Our institute works with signals coming from the staff; however, the system works top down.”* (QC7) In contrast to most directors, who decided to introduce the new QS and were involved in the development of the QS, QCs experienced the system as being imposed on them: *“Is it a system we introduce because we want to improve ourselves? Or is it a system that is imposed centrally”* (QC3). Therefore, not all QCs accepted the system; they looked at it as something with which they had to comply.

The strategies could not prevent the following bottlenecks at this stage: (1) participants had doubts about the credibility of the QS, (2) the approach did not fit every institute, and (3) the QCs felt the QS was imposed on them. These factors made it difficult for the system to be accepted in its entirety.

## Change

The introduction of the system involved many deadlines, to ensure that all institutes would take the different steps at the same time. While the QCs were prepared to work with the QS and felt supported, most also experienced time pressure to meet the deadlines and mentioned the many tasks that had to be performed. QCs felt there was not much room to deviate from the schedule and stated that it was a stressful period. Most directors experienced this more positively.

The participants experienced talking and thinking about quality both nationwide and at individual institutes as positive and stimulating. The results of the QS that participants mentioned were the following: introducing the system provided an opportunity to discuss quality, to look at the organization from a wider perspective, and to tighten the local policy. Notwithstanding these benefits, most participants mentioned that their institute invested more than they benefited from the system. They also stated that concrete results were lacking: *“The audit hasn’t brought us new insights, although the investments were very large; we expected to benefit more from it.” (D3).*

The barriers in the fourth stage that we found were: (1) a lack of flexibility, (2) time pressure — especially for the QCs, and (3) that the participants felt they had made considerable investments while the institutes had, so far, experienced a lack of new insights and concrete results.

## Consolidating change

After the firm deadlines, attention for the QS faded. At some institutes, the director and the QC had a clear and shared vision of the position of the system in the organization. This was helpful for keeping the system alive and for integrating the QS. However, most participants agreed that it was hard to integrate the QS into the organization. The national QC (component 4 of the implementation strategy) could play an important role in supporting the institutes with developing and implementing the improvement plans after the audit. Although participants mentioned they had faith and high expectations of the QC, so far she had not been actively involved.

## Discussion

This study aimed to gain more insight into the effect of the implementation strategy of a QS in a postgraduate medical specialty training. The four main components of the implementation strategy were (1) involvement of the directors of training institutes in the development of the whole system (content and process), (2) a web-based professional supportive system including a comprehensive manual, (3) a coordinated training programme, and (4) a national quality coordinator to support the institutes.

The results indicate that the implementation strategy was successful in preparing the institutes, helping the participants understand the potential benefits of the QS, completing necessary data collection in time, and creating a sense of togetherness in this process. Introducing common deadlines for data collection for all institutes enhanced peer pressure, and the participants indicated they found it stimulating to do this collectively. Our results, therefore, confirm previous findings that peer pressure and a sense of togetherness can contribute to an effective implementation: working together can enhance confidence and motivation, and prevent isolation [12, 15, 19].

Accepting the QS after the introduction, however, appeared to be difficult. This might be due to two factors: the perceived credibility of the new QS and the way the QS suited the local situation of the participating institutes. During the development of this system [17], we already observed that stakeholders doubted that the system was appropriate for measuring quality, and in this study we again observed that the participants were not convinced of this point. However, the literature reports that the people involved in a change-process have to feel the innovation is needed and appropriate [12, 16]. Consequently, we suggest that paying attention to the appropriateness and benefits of a QS for individual institutes and local contexts is important for the introduction of a shared QS.

QS acceptation may also have been difficult because quality coordinators (QCs) felt that the QS was imposed on them and that their investment in the system outweighed the benefits. We suggest it might be advisable to involve QCs and other staff - not just staff representatives - in the development of a QS. This may help build a broader base of support, which is likely to positively affect the acceptation of the QS. The literature confirms that a lack of ownership among staff is one of the biggest challenges in implementing an innovation [12]. Previous studies have also shown that the lack of concrete results is often a reason for an unsuccessful implementation [16, 20]. Our results suggest that the participants experienced the investments in the QS as much larger than the benefits. However, quality improvement has been shown to take between 5 and 10 years to achieve breakthroughs in continuous improvements in organization cultures [21]. Therefore, it can be helpful to be transparent about anticipated absences of short-term effects so that staff members can adjust their expectations.

Institutes agreed that integrating the QS was difficult. It seems that they needed more support in using the QS at their own institute. The chosen strategies, however, did not address this integration of the QS with local activities, cultures, and structures. More attention to the translation of the QS to practice therefore seems advisable. The literature also recognizes the importance of the translation of innovation to practice [22] and emphasizes the importance of the adaptability of an intervention to local circumstances. It appears to be difficult to keep the balance between interventions and local needs [15]. We suggest that institutes could benefit from the QS more optimally if they discuss what they need before launch in order to develop an action/implementation plan. An implementation plan is helpful in managing the process [21].

Our study showed some limitations and strengths. One strength was the use of the theoretical model/framework [16]. This was helpful for detecting facilitators and barriers in each of the different stages. A limitation was that we focused only on the directors and the QCs of training institutes. We did ask them about the perspectives of other staff, but we did not approach other staff directly. A second limitation is that we collected data early on in the process, which provides only limited insights into the last stage of the implementation process. However, had we delayed the data collection, people might have forgotten their early experiences with the system, and these experiences played an important role in the implementation process.

## Conclusions

In summary, this study shows the complexities of implementating a joint QS in the eight postgraduate medical specialty training institutes of the Netherlands, and it reveals several barriers to a successful implementation of such a QS. Practice points distilled from this study can be find in Table 4. More research on the implementation of a QS might be important as more knowledge of the effects of QSs can help convince staff and enhance the acceptance of a QS [12, 16]. Our predetermined implementation strategy focused on the preparation phase, and it gave little attention to the executing phase. The barriers we found mostly concerned the executing phase, in particular the connection with the local context. More focus on the context in which the institutes operate might have helped the integration of the QS at the separate institutes, and convergence with the local context may also enhance the sustainability of the QS.

**Table 4:** Practice points

• The implementation strategy was successful for the introduction of the quality system (QS) in the institutes.
• Creating a sense of togetherness and peer pressure can stimulate the use of the QS.
• In addition to directors and quality coordinators, staff also needs to be involved in the development and implementation of a QS.
• The appropriateness and benefits of the QS to local contexts needs to be clear.
• The relative absence of short-term effects should be communicated clearly.

## Abbreviations

D: Director
GEAR: Dutch acronym for Combined Evaluation Audit Round
GP: General Practitioner
QC: Quality Coordinator
QS: Quality System
WFME: World Federation for Medical Education
